# From Cyclones to Cybersecurity: A Call for Convergence in Risk and Crisis Communications Research

**DOI:** 10.1515/jhsem-2023-0067

**Published:** 2025-02-10

**Authors:** Ann Marie Reinhold, Ross J. Gore, Barry Ezell, Clemente I. Izurieta, Elizabeth A. Shanahan

**Affiliations:** Gianforte School of Computing, Montana State University Norm Asbjornson College of Engineering, Bozeman, USA; Virginia Modeling, Analysis & Simulation Center, Old Dominion University, Norfolk, USA; Department of Political Science, Montana State University, College of Letters and Science, Bozeman, USA; Pacific Northwest National Laboratory, Richland, USA

**Keywords:** hazard, disaster, communication, domain, discipline, scholarship

## Abstract

Effective risk and crisis communication can improve health and safety and reduce harmful effects of hazards and disasters. A robust body of literature investigates mechanisms for improving risk and crisis communication. While effective risk and crisis communication strategies are equally desired across different hazard types (e.g., natural hazards, cyber security), the extent to which risk and crisis communication experts utilize the “lessons learned” from scientific domains outside their own is suspect. Therefore, we hypothesized that risk and crisis communication research is siloed according to academic disciplines at the detriment to the advancement of the field of risk communications research writ large. We tested this hypothesis by evaluating the disciplinarity of 5,078 published articles containing risk and crisis communication keywords using a combination of simple descriptive statistics, natural language processing, and hierarchical clustering. Finding that the risk communication research is siloed according to disciplinary lexicons, we present our findings as a call for convergence amongst our risk and crisis communication scholars to bridge across our silos. In so doing, we will increase our ability to affect transformative change in the efficacy of our risk and crises messages across myriad hazard types – from cyclones to cybersecurity.

## 1 Introduction

Risk and crisis communication scholarship spans multiple hazard types, such as natural hazards (e.g., hurricanes (Millet et al. 2020); flooding (Raile et al. 2022; Shanahan et al. 2019); earthquakes (Herovic, Sellnow, and Sellnow 2020)); public health issues (e.g., pandemics (Berg et al. 2021)) and cancer (Julian-Reynier et al. 2003)); and national security (e.g., nuclear safety (Wu et al. 2019)) and cybersecurity (Zhang and Borden 2020)). A ubiquitous theme across these studies is a call for more effective strategies and approaches to risk and crisis communication that increase human and environmental safety and well-being and mitigate deleterious effects of a disaster. Whereas hazard case studies and issue topics vary, the goal to improve health and safety and reduce harmful effects remains unwavering.

Risk and crisis communication scholars have expended tremendous effort to improve the efficacy of messages (Munro et al. 2024). Yet, despite advances in risk and crisis communication, societies across the globe continue to experience increases in the frequency, magnitude, and cost of natural disasters, public health crises, and national security issues (De Wolf 2013; Garnett and Kouzmin 2007; United States Federal Emergency Management Agency 2022). Now is a critical time to identify possible areas of improvement in how risk and crisis communication scholars operate.

Existing risk and crisis communication strategies are pluralistic in theoretical grounding, resulting in no standard integrated approach (one notable exception is identification of the target audience). For example, some approaches include message construction (e.g., (Cho, Reimer, and McComas 2014; Reinhold, Munro, et al. 2023; Reinhold, Raile, et al. 2023)) others focus more on processes (e.g., (Coombs 2021)). Some approaches invoke a mental models approach (e.g., (Morgan 2002)) while others mass communication theory (e.g., (Austin et al. 2017)). While all meritorious in and of themselves, as a whole, they create a primordial soup of communication strategies and approaches through which practitioners must wade. In contrast, a convergent approach, by definition, “intentionally brings together intellectually diverse researchers to develop effective ways of communicating across disciplines” that ultimately result in the emergence of new frameworks.1
https://beta.nsf.gov/funding/learn/research-types/learn-about-convergence-research#definition



Additionally, how we, as risk and crisis communication scholars, approach developing best practices and strategies may inadvertently be imposing our own disciplinary structural challenges upon ourselves. Educational disciplines are defined by a canon of knowledge on a topic; theories and concepts; ontological and epistemological assumptions (what knowledge is and how it is discovered); methodologies, techniques, and procedures, and methods; lexicons; and some institutional manifestations (e.g., academic departments) (Dressel and Mayhew 1974; Krishnan 2009; Squires 1992). In turn, researchers trained in a particular discipline are often tethered to specific journals that share the tenants of their discipline. In this study, we explore the extent to which the gap between disastrous effects of hazards and advances in risk and crisis communication may be explained by our disciplinary silos and reward systems to publish in disciplinary journals. While we fully embrace the excellence of discoveries found in individual studies, the objective of this study is to explore whether we, as risk and crisis communication scholars, have imposed our own structural challenges upon ourselves.

To better understand if these structural challenges exist, we need to take stock of the state of this scholarship and develop some basic metrics that allow us to understand how the field of research has developed. These metrics include (i) the rate of risk and crisis communication publications over time, (ii) the ratio of these scholarly publications by journal outlet, and (iii) the distribution of these publications according to STEM, social science, and humanities domains. We predicted that (i) the field of risk and crisis communications – as indicated by numbers of publications – has grown rapidly over the last two decades, (ii) publications are widely distributed across outlets, and (iii) publications are largely siloed according to the domain of research (STEM, social science, humanities).

For this investigation, we conducted an extensive literature search of risk and crisis communication publications from 2002 to 2020. We assessed prediction (i) by plotting numbers of publications and outlets on an annual basis; prediction (ii) by evaluating the ratio of the number of publications to the number of publication outlets on an annual basis. We assessed prediction (iii) using natural language processing to assign the “disciplinarity” of each publication; we then performed a cluster analysis to investigate the extent to which risk and crisis communications research is siloed, and if siloed, how so.

## 2 Materials and Methods

We assessed each of the three predictions in two separate – but parallel – analyses. Specifically, we analyzed publications from all outlets and the top 30 outlets separately. Herein, we use the “top 30 outlets” to describe the 30 outlets containing the most publications for each year of the study period; i.e. some of these outlets change from year to year. We conducted these parallel analyses because we wanted to evaluate the publication patterns across the entire literature *and* for only the most common risk and crisis communications outlets.

**Figure 1:**
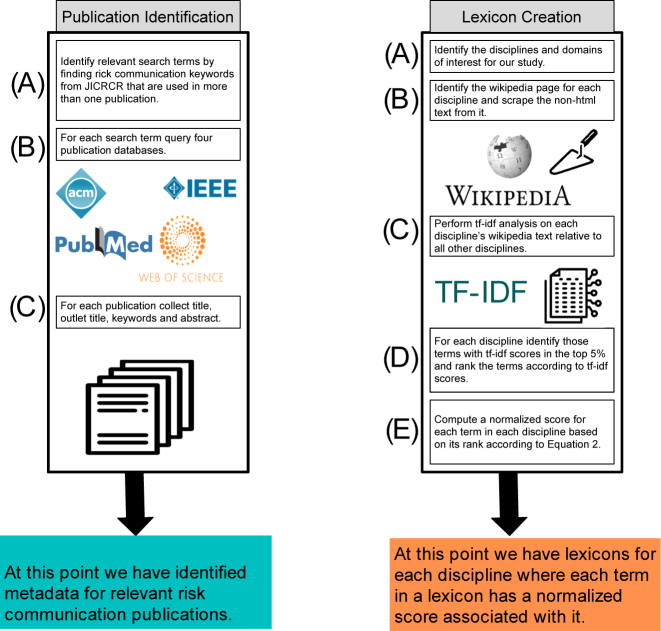
The processes of identifying publications and creating lexicons for disciplines. Letters indicate steps of each process.

To conduct these analyses, we identified a corpus of publications related to risk and crisis communication, and constructed a lexicon to quantify the extent to which each discipline is reflected in a publication. An overview of the process for identifying publications and creating lexicons is shown in Figure 1 and expanded below, beginning with how we identified relevant risk and crisis communications publications.

### 2.1 Publication Identification

#### 2.1.1 Compilation of Search Terms

Our discipline quantification process began with identifying a set of relevant risk communication publications. To assemble this corpus of publications, we first needed to compile a list of search terms (List 1).

These search terms were collected by finding all the keywords used in the publications of every issue of *The Journal of International Crisis and Risk Communication Research* (JICRCR)2
https://stars.library.ucf.edu/jicrcr/
 that matched the following regular expression:

(1) (risk)|(crisis)|(hazard)|(communica)|(prepar)

We retained all search terms that were present as keywords in two or more publications within JICRCR.

#### 2.1.2 Collection of Publication Metadata

We queried four different publication databases for each search term in List 1. These four databases were the Association for Computing Machinery Digital Library,3
https://dl.acm.org/
 PubMed,4
https://pubmed.ncbi.nlm.nih.gov/
 Web of Science,5
https://www.webofscience.com/wos
 and IEEE Xplore.6
https://ieeexplore.ieee.org/
 For each publication returned from each search term query, we cataloged the following publication metadata (if it was provided by the database): Title, Outlet Title, Abstract, Keywords and Date.

### 2.2 Publications Versus Outlets

We investigated the ratio of publications to outlets across the entire corpus of publications and on a subset of the corpus that contained only the top 30 outlets publishing risk and crisis communications research (i.e. the 30 outlets that published the largest number risk and crisis communications articles).

For the entire corpus, we conducted a simple linear regression on the number of publications with risk and crisis communication keywords versus the number of outlets (e.g., journals, conference proceedings) on an annual basis. For publications from the top 30 outlets, we could not conduct a regression while holding the outlets constant; a regression cannot be conducted while holding the independent variable to a constant value (here, 30 outlets).

### 2.3 Discipline Lexicon

#### 2.3.1 Creation of Discipline-Specific Lexicons

To create discipline-specific lexicons, we first identified 25 different disciplines across three domains. Domains and disciplines were chosen based on the expert opinion of our team. The domains are Humanities, Social Sciences, and Science, Technology, Engineering and Math (STEM). The disciplines included in the Humanities domain are History, English Language, Religious Studies, Media Studies, Philosophy, and Journalism. The disciplines included in Social Sciences domain are Management, Business, Public Administration, Political Science, Policy, Economics, Communication, Sociology, Anthropology, Psychology, and Education. The disciplines included in STEM are Information Science, Computer Science, Engineering, Earth Science, Astronomy, Biology, Chemistry, and Physics.

For each discipline, we created a lexicon to enable us to quantify the extent to which the discipline is reflected in each publication. We call this measure the *disciplinarity* of a *publication*_*x*_ given a *discipline*_*y*_. The process for our lexicon creation for a given discipline was as follows:

– Identify the Wikipedia7
https://www.wikipedia.org/
 page describing the *discipline*_*y*_.
– Scrape the non-html text from the Wikipedia page for *discipline*_*y*_.
– Once the Wikipedia page for each *discipline*_*y*_ has been scraped:
  – Compute the term frequency-inverse document frequency (TF-IDF) scores for each non-html term on the Wikipedia page of *discipline*_*y*_ with respect to all other disciplines.
  – Identify the terms with TF-IDF scores in the top 5 % for *discipline*_*y*_.
  – Put the identified terms (those TF-IDF scores in the top 5 %) in rank order.
  – For the identified, rank-ordered terms for *discipline*_*y*_, produce a normalized score for each term according to Equation (2). In Equation (2), *i* reflects the rank of the term being scored and *n* reflects the rank of the term with the lowest TF-IDF score (i.e. final rank).

(2) $$\frac{\left(n+1\right)-i}{n}$$

We employed TF-IDF to quantify the uniqueness of terms in each Wikipedia page for each discipline with respect to the Wikipedia page for all other disciplines. Briefly, TF-IDF consists of two parts, TF (term frequency) and IDF (inverse document frequency). For a given term (*t*) and a given discipline (*discipline*_*y*_) from our set of disciplines (*disciplines*), it is defined by Equation (3).

(3) $$tf\left(t,{discipline}_{y}\right)*idf\left(t,disciplines\right)$$

In this study, TF quantifies the frequency of a specific term (*t*) in the scraped Wikipedia page of a discipline (*discipline*_*y*_) with respect to the total number of terms in the Wikipedia document for *discipline*_*y*_, counting each occurrence of the same term separately (*M*). The TF portion of the TF-IDF calculation is shown in Equation (4).

(4) $$tf\left(t,{discipline}_{y}\right)=\frac{f_{t,{discipline}_{y}}}{M}$$

In this study, IDF quantifies how unique a term (*t*) is to the scraped Wikipedia page associated with a discipline compared to all other scraped Wikipedia pages for other disciplines. IDF is the logarithmically scaled inverse fraction of the Wikipedia discipline documents that contain the term. It is obtained by dividing the total number of Wikipedia discipline documents (|*disciplines*|) by the number of Wikipedia discipline documents containing the term, and then taking the logarithm of that quotient. The IDF portion of the TF-IDF calculation is shown in Equation (5).

(5) $$idf\left(t,disciplines\right)=\log\frac{|disciplines|}{\#\;of\;occurences\;of\;t\;in\;all\;disciplines}$$

The TF-IDF scores within the lexicon of each discipline were ranked and normalized to control for potential bias from rare terms. The normalization process ensured each lexicon had terms within the same value range (0.0–1.0). In combination, these methodological decisions enabled every publication to be scored for every discipline without systemic bias.

### 2.4 Measurement of the Disciplinarity of each Publication

**Figure 2:**
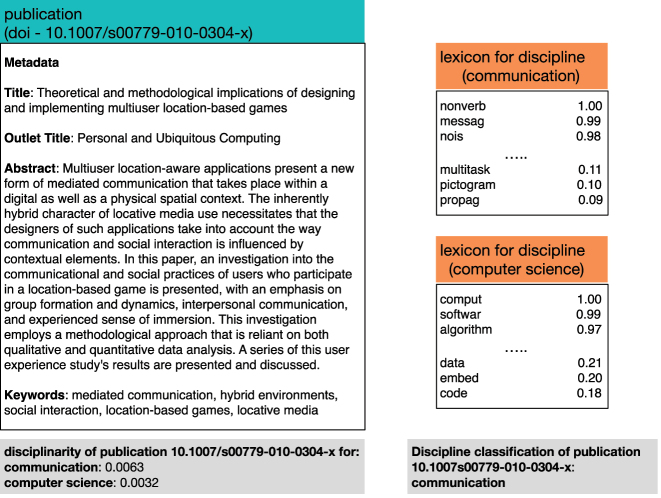
The disciplinarity scoring and discipline classification for an example publication.

Each publication was scored to determine its *disciplinarity*. Recall, *disciplinarity* is the extent to which a *publication*_*x*_ reflects *discipline*_*y*_. The computation of the disciplinarity of *publication*_*x*_ with respect to *discipline*_*y*_ is shown in Figure 2 and described as follows.

– The collected metadata (i.e. title, outlet title, abstract and keywords) associated with *publication*_*x*_ were concatenated into a single string. We refer to this as the metadata string for *publication*_*x*_.
– The metadata string for *publication*_*x*_ was scored with the normalized lexicon for *discipline*_*y*_. We refer to this score as the disciplinarity score of *publication*_*x*_ with respect to *discipline*_*y*_.
– The disciplinarity score of *publication*_*x*_ with respect to *discipline*_*y*_ was calculated as the sum of the normalized scores for the terms in the metadata string in *publication*_*x*_ that appeared in the lexicon for *discipline*_*y*_ divided by the number of total terms in the metadata string for *publication*_*x*_. If there were not any terms in the metadata string for *publication*_*x*_ that appeared in the lexicon for *discipline*_*y*_, then *publication*_*x*_ was given a disciplinarity score of 0 with respect to *discipline*_*y*_.

Thus, the belonging of a publication (*publication*_*x*_) to each discipline (*discipline*_*y*_) is the disciplinarity score for the publication with respect to each of the 25 disciplines.

### 2.5 Cluster Analysis

We conducted two hierarchical cluster analyses; one on the disciplinarity scores of all publications across all disciplines *and* another on the disciplinarity scores of all publications from the top 30 outlets across all disciplines. For each cluster analysis, we first scaled and centered the disciplinarity scores for all publications within each discipline because the normalized disciplinarity scores were highly variable among disciplines. We subsequently created a Euclidean distance matrix of those data and conducted hierarchical clustering with average linkage (i.e. unweighted pair group method with arithmetic mean [UPGMA]).

To fully understand the cluster analysis results, it is important to note that our approach to defining a lexicon for each discipline is independent and separate from our approach to classifying each publication. The lexicon creation relies on identifying and weighting terms that are unique to a discipline based on the entries of Wikipedia pages (not publications). Our approach to classifying the disciplinarity of publications uses the Wikipedia-based lexicons to quantify the probability that a publication belongs to each discipline. Each publication is then classified according to the disciplinarity scores. Using this approach, we next use the cluster analysis to highlight the extent to which publications classified into different disciplines are similar based on the probability that they belong to each of the other disciplines. 

### 2.6 Data Availability

Data and code to reproduce our analyses are available at the following DOI: 10.17632/9jcdz2k2tc.2. 

### 2.7 Threats to Validity

In this subsection we examine the threats to validity of this work as described by Campbell and Stanley (Campbell and Stanley 1963) and Wohlin et al. (Wohlin et al. 2012). Specifically, we discuss Conclusion Validity, Content Validity, Internal Validity, Construct Validity, and External Validity, as well as Reliability as described by Yin (Yin 2009). The evaluation of each of the threats to these specific types of validity are elaborated in the following bullets.

– *Content Validity* refers to how representative the selected measures cover the content of a study. A potential source of bias here is the chosen set of databases. While the chosen set of databases reflect collections of publications across a variety of disciplines, they do not represent the entire universe of publication databases. In addition, we chose to only include publications from 2002 through the end of 2020. This choice was made so that our work reflects the current risk and crisis communication landscape. However, it also means our work is not generalizable to other time periods.
– *Internal Validity* refers to the possibility of having unwanted causal relationships. The use of Wikipedia to create our discipline lexicons is a potential threat to internal validity. While it has become an established source of information, it only reflects a single description of each discipline and could have been modified by uninformed or nefarious parties, although this is unlikely to have occurred to an extent that it impacts our results.
– *Construct Validity* refers to the meaningfulness of measurements and the choices made regarding the selection of independent and dependent variables such that these variables are representative of theory. Our study hinges on the idea that publications and the language used therein provides a meaningful barometer of disciplinarity. Given our collective experiences and professional opinion, we do not anticipate this to be a meaningful threat to validity. Moreover, lexicon-based approaches to classifying sentiment, emotions, and domains are widely used (Bonta, Kumaresh, and Janardhan 2019). While these techniques to do not explicitly account for semantic meaning (herein, disciplinarity), they have been successful in providing insight into research questions that were previously difficult to answer. While several studies have applied semantic structures to improve lexicon-based approaches, they have done so with only modest (∼2–7 %) improvements to accuracy (Saif et al. 2014). Furthermore, because these semantic structures require semantic data to facilitate classifications they are more subjective and less generalizable than strictly lexicon-based approaches such as the one we apply here (Salloum, Khan, and Shaalan 2020). Thus, adding semantic structures to our lexicon-based approach may have resulted in minimal improvements to construct validity but would have reduced the replicability of this study and increased its complexity.
– *External Validity* refers to the ability to generalize results. As in most case studies, the ability to generalize results is limited due to the inability to randomly sample or to randomly assign subjects to groups. However, it can be argued that a narrower definition of external validity – ecological validity – the extent to which these findings approximate similar situations in other real-world environments, is likely to hold. We expect that other studies would find similar results if additional databases and journals were scoured, as we have searched four large, reputable databases that span domains and disciplines.
– *Reliability* refers to how dependent the process is on the researchers. We compiled the list of disciplines and domains based on literature review and expert opinion. Different researchers may devise slightly different discipline categories. While subtle differences may occur, we would not expect the magnitude of differences in discipline categories to alter the results presented here substantively.

## 3 Results

### 3.1 Prediction (i)

The field of risk and crisis communication research has grown substantially over the last two decades in line with what we predicted. While this growth is nonstationary, it is significant. These results indicate that the saliency of the scholarship on risk and crisis communication is high.

**Figure 3:**
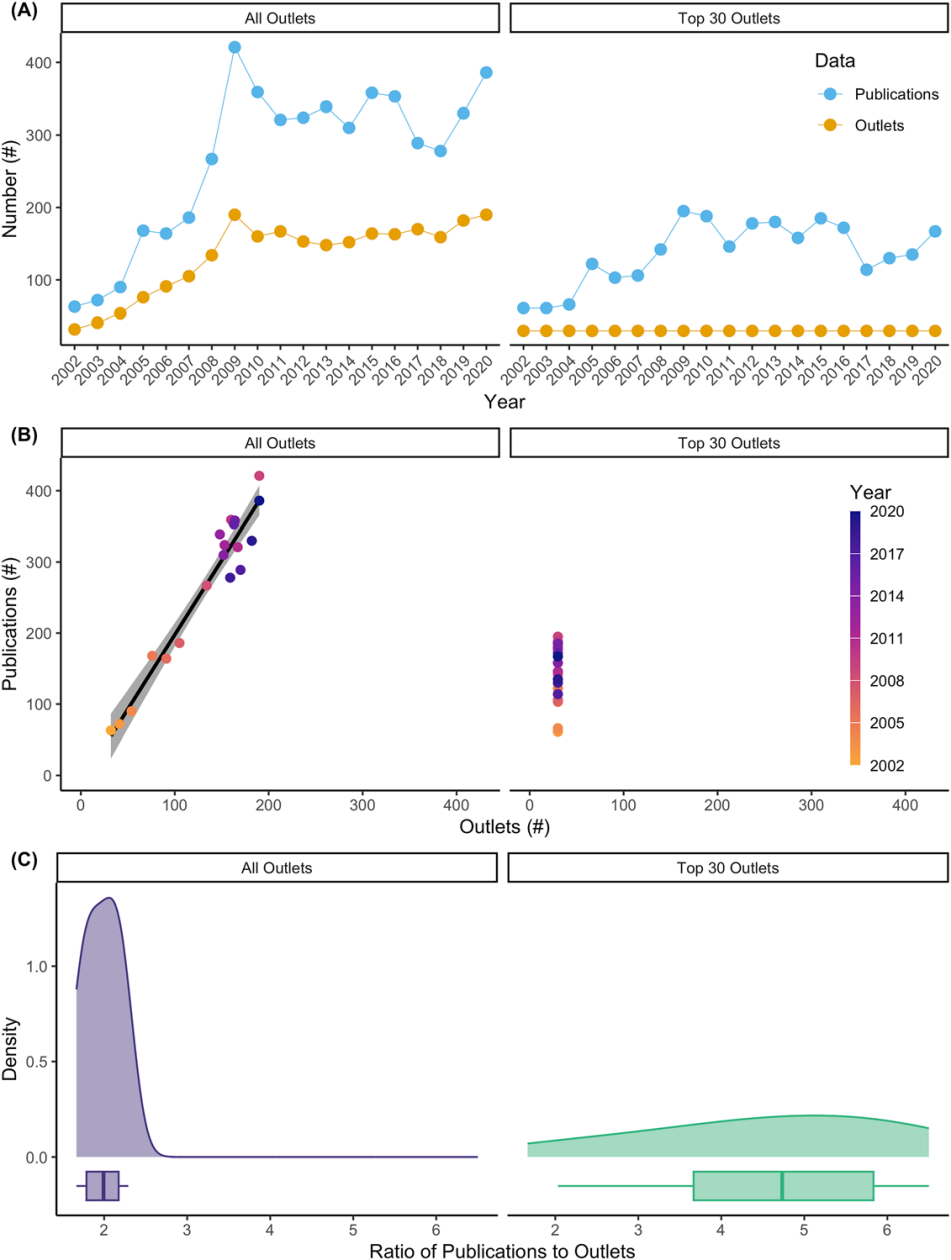
Publication patterns in risk and crisis communications research. Figure contains two columns; left column contains results from the entire corpus of publications whereas right column contains results for the portion of the corpus containing publications originating from only the top 30 outlets. (A) Numbers of publications (blue) and outlets (gold) with risk and crisis communication keywords versus year. Note that the number of outlets is held to 30 intentionally in the right-hand panel. (B) Number of publications with risk and crisis communication keywords versus the number of outlets (e.g., journals, conference proceedings). Point color corresponds to year of publication in an outlet. Thick black line depicts result of simple linear regression of publication number versus outlet number on an annual basis; grey shading indicates 95 % confidence band. Note that the number of outlets is held to 30 intentionally in the right-hand panel. (C) Density plots and boxplots depicting the ratios of publications to outlets for the entire corpus (left) and for the portion of the corpus containing publications from the top 30 outlets (right).

**Figure 4:**
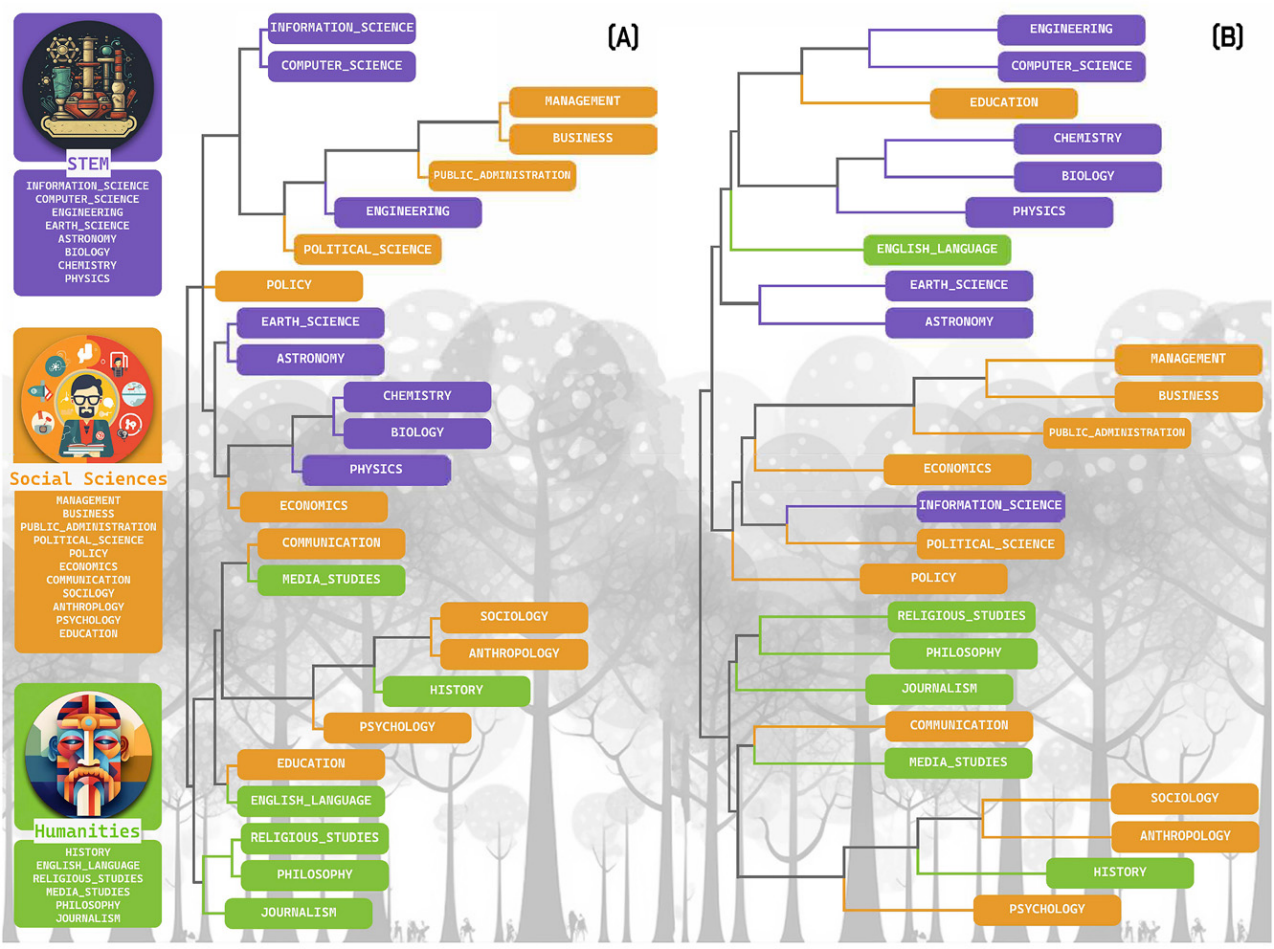
Structure of risk and crisis communication publications according to discipline. Hierarchical clustering of literature on risk and crisis communications with discipline color mapping to domain (STEM, purple; social sciences, orange; humanities, green). Panel (A) contains results from the entire corpus of publications whereas panel (B) contains results for the portion of the corpus containing publications originating from only the top 30 outlets. Graphic design by Bratislav Cvijetić.

#### 3.1.1 All Outlets

The volume of risk and crisis communications publications indicates that this is an active area of research. Over the course of the study period (2002–2020) alone, we identified 5,078 publications containing risk or crisis communication keywords. The growth of this field of research is not stationary (Figure 3A). Rather, the development of this literature has two distinct periods. During the period from 2002 to 2009, the field experienced sustained growth. During the period from 2010 to 2020, the volume of publications remained high but has stabilized.

From 2002 to 2009, both the number of publications and the number of outlets increased dramatically. Using the endpoints of this period to assess growth, the number of publications increased by a factor of 6.7 from 63 to 421 between 2002 and 2009; the number of outlets increased nearly six-fold from 32 to 190 over the same period. From 2010 to 2020, the number of publications averaged 331.5 with limited fluctuation (standard deviation [SD] = 32.0). The number of outlets was similarly stable during this period, with an average of 164.4 outlets (SD of 12.7).

#### 3.1.2 Top 30 Outlets

In the top 30 outlets, we identified 2,609 topical publications over the course of the study period. The rise in risk and crisis communications publications is evident from 2002 to 2009 for the top 30 outlets. Using the endpoints of this period to assess growth, the number of publications increased by a factor of 3.2 from 61 publications in 2002 to 195 publications in 2009. From 2009 to 2020, the publication rates remain high, although variability in publication rates exist. During this period, the average number of publications was 159.4 with an associated SD of 24.8.

### 3.2 Prediction (ii)

Our prediction was that the scholarship on risk and crisis communications was highly disbursed. We find that many outlets exist for scholarly publications on risk and crisis communications research. However, each of these outlets published relatively few risk and crisis publications each year. This result indicates that the literature on risk and crisis communications is widely distributed throughout the literature.

#### 3.2.1 All Outlets

On average, each outlet published relatively few articles. Each outlet contained a median of 2.0 risk and crisis communications publications across the study period (Figure 3C). This was consistent throughout the study period, despite the differences in rates of publications from 2002 to 2009 and 2010–2020 (*slope* = 2.1, *t-value* = 15.9 and *P-value* < 1e^−11^ on 1 and 17 degrees of freedom from a linear regression; Figure 3B).

#### 3.2.2 Top 30 Outlets

In the top 30 outlets, each outlet contained relatively few publications. Each outlet contained a median of 4.7 publications across the study period (Figure 3C). Rates of publications in the top 30 outlets was lower in 2002–2004 (median = 2.0 publications per year; SD = 0.10) than from 2005 to 2020 (median = 5.1 publications per year; SD = 1.0).

### 3.3 Prediction (iii)

Our prediction was that the academic publications from disciplines within the domains of the Humanities, Social Sciences, and STEM fields would fall into three distinct clusters. That is, we predicted that publications with language strongly associated with a discipline in the humanities would all share one common root node whereas publications with strong Social Science language would originate from another common root node, and publications with strong STEM language would all be associated with yet another distinct root node. Our results did not adhere to this prediction entirely; however, the cluster analysis did reveal distinct lexicons that are indicative of silos. That is, closely related disciplines clustered together both within and across domains (Figure 4).

#### 3.3.1 All Outlets

Publications with strong STEM language clustered in the upper branches (Figure 4A, purple) whereas publications with strong humanities language clustered in the lower branches (Figure 4A, green). This clustering indicates that publications with high scores for STEM language had low scores for humanities language and vice versa. Only disciplines within the social science domain were dispersed among the upper (STEM-heavy) and lower (humanities-heavy) branches of the dendrogram, but even their positioning indicates some degree of siloing.

Closely related disciplines clustered together, reflecting ontologies among disciplines. Within the STEM domain for instance, the disciplines of information science and computer sciences clustered together, as did earth science and astronomy, and chemistry, biology, and physics. Likewise, within the humanities domain, religious studies and philosophy clustered together. Among the domains of the humanities and social sciences, closely related disciplines also clustered together; media studies clustered with communication, and history clustered with sociology, anthropology, and psychology.

#### 3.3.2 Top 30 Outlets

The results of the cluster analysis for the publications from the top 30 outlets (Figure 4B) were similar to the results from all outlets (Figure 4A). Publications with strong STEM language generally clustered in the upper branches. Publications with strong humanities and social science disciplinarity scores generally grouped together in the lower branches.

Only publications with high disciplinarity scores for information science (a STEM discipline) were found in the lower branches (among the Social Sciences and Humanities disciplines). More specifically, information science and political science publications grouped together, indicating a similar lexicon for these two disciplines in publications from the top 30 outlets.

Only publications with high education and English language disciplinarity scores were grouped in the upper branches. The placement of the education and English language scores in the upper branches indicates that the lexicons in these publications are more similar to the lexicons in the STEM publications than the other humanities and social sciences publications.

As with the cluster analysis of publications from all outlets (Figure 4A), we see evidence of disciplinary silos in the results from the top 30 outlets (Figure 4B). As with the whole corpus analysis (Figure 4A), management, business, and public administration publications grouped together. Likewise, STEM publications with high disciplinary scores for chemistry, biology, and physics grouped together, as did humanities papers with high disciplinarity scores for religious studies and philosophy. Closely related disciplines also clustered together across the domains of humanities and social sciences; media studies clustered with communication again, and history clustered with sociology, anthropology, and psychology again.

## 4 Discussion

Our current work provides an important baseline for taking stock of the field of risk and crisis communications research. The benefits of this work are threefold. We have: (1) characterized the development and state of the scholarship on risk and crisis communication research, (2) identified metrics for this characterization, and (3) developed benchmarks for assessing the extent to which this field of research shifts towards (or away from) siloing in the future.

With respect to the state of the field, our team’s collective experience working across the research areas of natural hazards, viral spillover, and cybersecurity led us to posit that the literature on risk and crisis communication was highly distributed and disconnected. We find that although risk and crisis communication scholars have expended considerable effort to publish a large volume of studies, these scholars lack a common vocabulary for convergent research advances. This lack of a common vocabulary is indicative of silos, which we characterize herein (see Section 3.3).

The ontogeny of disciplinary silos is understandable given the diversity of risk and crises that this literature addresses. However, breaking down these silos is an opportunity to advance the scholarship because risks and crises occur across disciplines and domains. Consequently, funding agencies (e.g., National Science Foundation) are supporting convergent8
https://beta.nsf.gov/funding/learn/research-types/learn-about-convergence-research#definition
 research and innovation ecosystems. We suggest that convergent research will enable advancements that transcend domains. We posit that convergence in risk and crisis communications research includes: a shared understanding of domains and disciplines; concepts that cut across case study idiosyncrasies (Reinhold et al. 2023); and a domain-agnostic lexicon. The resulting approach will be adopted more generally by communication scholars and practitioners (in line with the successful NSF-funded CONVERGE Facility (Peek et al. 2020)).

With respect to the metrics and methodology we employed to quantify the current state of convergence in the field of risk and crisis communication, the same metrics can be used to measure the state of the field in the future. We selected metrics that are intentionally straightforward, repeatable, and parsimonious to facilitate their reuse. The use of third party, objective materials to identify search terms and define discipline lexicons, is meant to promote clear and simple assessment of the field in an unbiased manner. We do not suggest that these metrics are an end; rather, they are an initial step towards characterizing the state of convergence of the scholarship in the field. In future work, we will explore using bibliometrics to measure the impact of research within and across silos. This strategy will quantify the extent to which researchers within silos are aware of and acknowledging one another’s work regardless of whether the language used in their publications is domain agnostic or domain specific.

With respect to the development of benchmarks for assessing how the field of risk and crisis communication progresses, our approach here can be used in the future to measure how the field is growing and the extent to which our scholarship is converging (or not). It is almost certain that there will be a large increase in publications associated with the risk and crisis messaging during the COVID-19 pandemic; our preliminary research indicates that this is so. What is less certain is where these articles will be published and if they will be widely disbursed throughout the literature. As those data on publication rates become available, the approach and metrics utilized here provide a foundation for assessing exactly how the field is evolving – towards or away from a convergent lexicon. We now have a prototype frame for assessing improved convergence or increased siloing. For instance, if the field is moving towards convergence, we expect to see the ratio of publications to outlets to increase (Figure 3B and C); also, we would expect to see the cluster analysis with less depth and more mixing among disciplines across domains such that, e.g., disciplines within the humanities domain are well intermixed with disciplines in the STEM domain (Figure 4).

The need for taking stock of the field of risk and crisis communications research and developing these benchmarks is timely and perhaps even overdue. Assessing our current state is imperative as we take steps towards convergence (Adams, Evans, and Peek 2023; Adams et al. 2022; Peek et al. 2020) and measure the impact of those steps. We view this as a research imperative because we, the risk and crisis communication scholars, face substantive challenges as we seek to employ best practices in risk and crisis communications research, including structural obstacles that exist beyond our control.

In addition to our disciplinary silos, other structural obstacles are also present challenges. First, we live in an information age where images, words, and ideas are widely available and quickly disseminated, often resulting in information overload, i.e. when the level of information is greater than information processing capacity (Roetzel 2019). Thus, the importance of identifying a target audience is now coupled with competition for attention in the cacophonous environment of an endless information stream. Second, unequal information access, challenges in intergovernmental coordination, concerns of trust and credibility (Cairns, De Andrade, and MacDonald 2013), and poor numeracy (Rakow, Heard, and Newell 2015) present formidable challenges for risk and crisis communicators to surmount. Third, we now live in the Anthropocene, where human activities are impacting the environment, resulting in novel disaster events (Keys et al. 2019) and subsequently leading to more complex, interwoven, and multifaceted hazard scenarios.

Risk and crisis communications scholars have minimal power to affect change when it comes to information overload, structural challenges, and implications of living in the Anthropocene. However, we do have agency over how we conduct our studies, where we disseminate them, and how we interact with one another. Breaking down silos and building bridges amongst us will enable advancements in risk and crisis communications to better achieve the broader impacts that the scholarly community desires: to increase human and environmental safety and well-being, and mitigate the deleterious effects of a disaster. We share a common vision with risk and crisis communication scholars writ large: enhancing human and environmental safety with our work. Therefore, we assert that there is great value in researching ways to break down these silos and build bridges across scientific disciplines and domains.

**List 1 Search Terms Used to Assemble Corpus of Publications**: Communication Management, Crisis Communication, Crisis Perception, Crisis Preparedness, Crisis Tolerance, Crisis Actor, Crisis and Emergency Risk Communication, Crisis Communication Theory, Crisis Emotions, Crisis Framing, Crisis Influence, Crisis Management, Crisis Response, Crisis Type, Crisis Typology, Disaster Communication, Disaster Perception, Disaster Preparedness, Disaster Tolerance, Emergency Risk Communication, Hazard Communication, Hazard Perception, Hazard Preparedness, Hazard Tolerance, Health Communication, Health Crisis, Health Risk, Instructional Risk and Crisis Communication, Interpersonal Communication, Legal Crisis, Megacrisis, Organizational Crisis, Perceived Crisis Severity, Refugee Crisis, Risk Communication, Risk Perception, Risk Preparedness, Risk Tolerance, Risk and Crisis Communication, Situational Crisis Communication Theory, Social-Mediated Crisis, Social-Mediated Crisis Communication, Sports Communication, Stakeholder Communication, Strategic Communication, Transparent Organizational Communication, Water Crisis.
